# Lack of Evidence for Crimean–Congo Hemorrhagic Fever Virus in Ticks Collected from Animals, Corsica, France

**DOI:** 10.3201/eid2805.211996

**Published:** 2022-05

**Authors:** Vincent Cicculli, Apolline Maitre, Nazli Ayhan, Stevan Mondoloni, Jean-Christophe Paoli, Laurence Vial, Xavier N. de Lamballerie, Remi Charrel, Alessandra Falchi

**Affiliations:** Laboratoire de Virologie, Université de Corse–Institut National de Santé et de la Recherche Médicale, Corte, France (V. Cicculli. A. Maitre, A. Falchi); Unité des Virus Emergents, Aix Marseille Université, Marseille, France (V. Cicculli, N. Ayhan, X.N. de Lamballerie, R. Charrel);; Laboratoire de Recherches sur le Développement de l’Élevage, Institut National de la Recherche pour l’Agriculture, l’Alimentation et l’Environnement, Corte (J.-C. Paoli);; Campus International de Baillarguet, Montpellier (L. Vial);; Parc Naturel Régionale de Corse, Corte (S. Mondoloni)

**Keywords:** Crimean–Congo hemorrhagic fever, ticks, tickborne disease, Nairovirus, Corsica, France, viruses, vector-borne infections, zoonoses

## Abstract

In Corsica, France, 9.1% of livestock serum samples collected during 2014–2016 were found to have antibodies against Crimean–Congo hemorrhagic fever virus (CCHFV), an emerging tickborne zoonotic disease. We tested 8,051 ticks for CCHFV RNA and *Nairovirus* RNA. The results indicate that Corsica is not a hotspot for CCHFV.

Crimean–Congo hemorrhagic fever (CCHF) is a tickborne zoonotic disease that is characterized by hemorrhagic fever and can progress from mild, nonspecific signs to a severe and fatal hemorrhagic disease. The CCHF virus (CCHFV) is an enveloped, segmented, negative-sense, single-stranded RNA member of the family Nairoviridae, genus Orthonairovirus. CCHFV has been detected in >35 species of ticks worldwide, among which ticks belonging to the genus *Hyalomma* are the primary vectors in humans and wild and domestic animals (*1*). Humans are infected through tick bites and direct contact with infected blood and body fluids during occupational exposure (e.g., farming, slaughtering, and medical and nursing care). 

CCHF is endemic in Africa, Asia, and the Balkan region (*2*). In Western Europe, autochthonous human cases were reported only in Spain, where CCHFV was identified in *H. lusitanicum* ticks (*3*). In Corsica, a French Mediterranean island, 9.1% of livestock (i.e., cattle, goats, sheep) serum samples contained CCHFV-specific IgG during 2014–2016 (*4*). Entomologic surveys revealed that the *H. marginatum* tick, a vector of CCHFV, was present in Corsica (*5*).

## The Study

To assess whether CCHFV circulates in Corsica, we collected 8,051 ticks from wild and domestic animals in selected sites on the island during 2016–2020 (Table, Figure). These 8,051 ticks included 7,156 ticks taken from 3,674 domestic animals and 895 ticks taken from 188 wild animals. They consisted of 4,177 *Rhipicephalus bursa* (51.8%), 2,386 *H. marginatum* (29.6%), 839 *Dermacentor marginatus* (10.4%) and 282 *H. scupense* (3.5%) ticks. We identified ticks at the species level by using a pictorial guide and confirmed morphologic identification by using sequencing of mitochondrial 16S rDNA (*5*). We then pooled up to 10 ticks per pool on the basis of developmental stage (nymphs, nonengorged females, and male adults) and host (Table). Pools, containing an average of 2.5 ticks (range 1–10 ticks) were crushed in phosphate-buffered saline with TissueLyser II (QIAGEN, https://www.qiagen.com) at 5,500 rpm for 3 min. We spiked each pool before extraction with a predefined amount of MS2 bacteriophage to monitor the subsequent steps (nucleic acid extraction, reverse transcription, and PCR amplification) and to detect the presence of inhibitors and enzymatic reactions as described (*6*). We performed DNA extraction by using QIAcube HT and a QIAamp cador Pathogen Mini Kit (QIAGEN), according to the manufacturer’s instructions. We eluted DNA in 150 μL of buffer and stored at –20°C. We tested each pool for the presence of CCHFV RNA by using a real-time, reverse transcription PCR (*7*) and the presence of *Nairovirus* RNA by using a pangeneric reverse transcription PCR (*8*).

**Table:** Ticks collected, by host, number of ticks, and number of tick pools, in a study of Crimean–Congo Hemorrhagic fever virus in ticks from wild and domestic animals, Corsica, France, 2016–2020

Host and tick species	No. ticks	No. tick pools
Cattle, n = 1,211		
* Rhipicephalus bursa*	3,413	818
* Hyalomma marginatum*	1,343	475
* H. scupense*	282	96
* Boophilus annulatus*	130	47
* Ixodes ricinus*	85	33
* H. punctata*	14	10
* R. sanguineus*	96	32
* Dermacentor marginatus*	2	2
Total	5,365	1,513
Horses, n = 201		
* H. marginatum*	1,026	247
* R. bursa*	637	135
* R. sanguineus*	27	10
Total	1,690	392
Wild boar, n = 182		
* D. marginatus*	837	222
* H. marginatum*	13	7
* R. bursa*	9	6
* I. ricinus*	13	5
* R. sanguineus*	1	1
Total	873	241
Sheep, n = 773		
* R. bursa*	101	93
Total	101	93
Deer, n = 4		
* R. bursa*	9	4
* H. marginatum*	4	1
Total	13	5
Mouflon sheep, n = 2		
* R. bursa*	8	5
* I. ricinus*	1	1
Total	9	6
Overall	8,051	2,250

**Figure:**
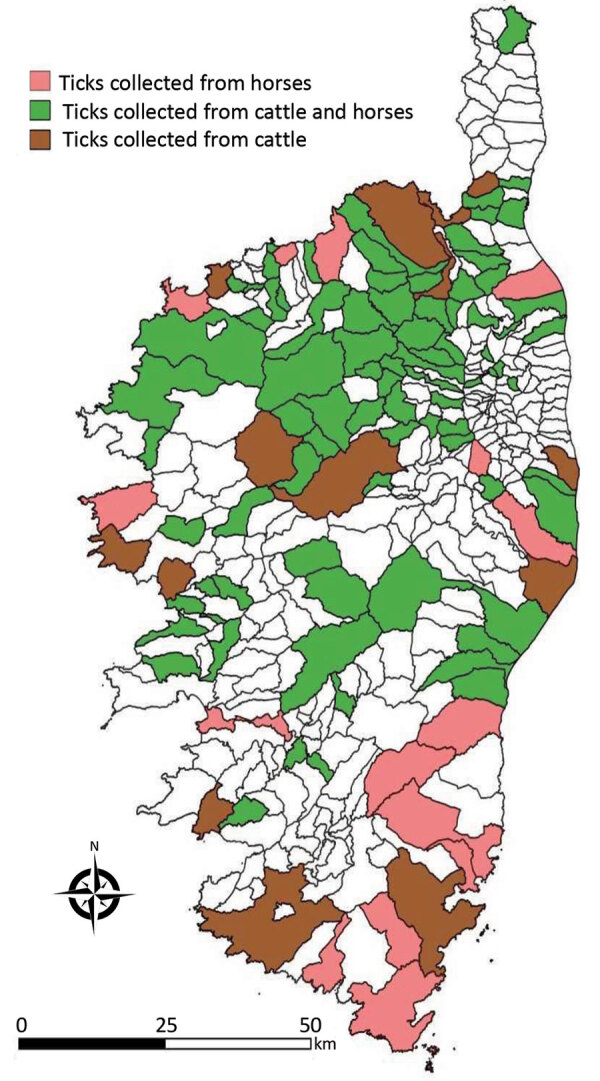
Locations of tick collection sites (for cattle and horses) for a study of Crimean–Congo Hemorrhagic fever virus in ticks from wild and domestic animals, Corsica, France, 2016–2020.

We detected neither CCHFV RNA nor *Nairovirus* RNA in the 8,051 ticks. The absence of CCHFV or *Nairovirus* RNA was not attributable to technical problems or presence of inhibitors, which were ruled out by MS2 bacteriophage monitoring. Moreover, we detected viral RNA corresponding to new tickborne *Phleboviruses* in 40 samples (5%) and *Flavivirus* in 7 samples (0.9%); these samples remain under investigation, and results will be reported after detailed characterization.

## Conclusions

We considered whether CCHFV RNA was not detected because of a low minimum infection rate (MIR) that a larger number of ticks would have been required. We calculated the theoretical power that could be achieved by using the number of ticks obtained in our study. On the basis of an expected CCHFV prevalence (*P*) of ≈0.2% and a pool size (*k*) of 2 ticks, a total of 7,676 ticks have to be tested for a prevalence estimation with a 95% CI and a precision (*d*) set at +0.001 because the disease prevalence is <0.1 (10%) (*9*). Thus, with a sample of 8,051 ticks, we were able to detect a prevalence of >0.2%.

The rate of CCHFV-infected ticks in countries in Europe with enzootic foci ranges from 0.50% to 3.70% among *Hyalomma* spp. ticks (2.8% [44/1,579 *H. lusitanicum* ticks] in Spain, 3.7% [6/161 *H. marginatum* ticks] in Bulgaria, and 0.5% [1/199 *H. marginatum* ticks] in Kosovo) and from 1.5% to 6.2% among *Rhipicephalus* spp. (1.5% [2/123 *R. sanguineus* ticks] in Bulgaria and 6.2% (8/130 *R. bursa* ticks] in Kosovo) (*10*). Other studies conducted outside of Europe have largely reported MIR values >0.2% among ticks: 0.71% in South Africa (1.6% [15/914] *H. truncatum* and 0.2% [2/1,149] *H. rufipes*) (*11*); 2.6% in Mauritania (39/1,517 *Hyalomma* spp.) (*12*); 3.8% (20/525 *Hyalomma* spp.) in Pakistan (*13*); and 51.5% (103/200 *H. marginatum*) in Turkey (*14*). These studies were conducted during the past 5 years using methods comparable to those of our study. The number of *Hyalomma* (n = 2,682) and *Rhipicephalus* (n = 4,177) ticks that we tested are much higher than reported in these previous studies. Therefore, our study would have been able to recognize CCHFV presence for a prevalence >0.2%, which is 10 times lower than the lowest overall prevalence value reported to date in countries where CCHFV is present: 2.1% (95% CI 1.3%–2.9%) according to a recent meta-analysis (*10*). Furthermore, another study addressing the presence of CCHFV RNA in *Hyalomma* spp. ticks (362 *H. marginatum* and 135 *H. scupense*) and *Rhipicephalus* ticks (n = 518) collected in 2014 from domestic and wild animals in Corsica also provided only negative results (*15*). In all countries where CCHF cases are described, the observed MIR of ticks is >2.5 times higher than the detection limit in our study (0.2%). Another argument that strongly supports the contention that the lack of detection of CCHFV or *Nairovirus* RNA was not caused by technical problems is based on the consideration that the protocol used in this study enables the detection of a wide variety of different CCHFV strains, a fact that confirms the accuracy of the results (*7*,*8*).

Recent studies to determine whether CCHFV is present in Corsica and to what extent it is a threat for human populations provide contrasting data. On one hand, tick species that are able to transmit CCHFV are present and widely distributed, and a serologic study based on ELISA screening and neutralization test for confirmation supports the presence of CCHFV or an antigenically related agent. On the other hand, the absence of detection of CCHFV RNA (or an antigenically related agent) in a large number of ticks, together with the absence of a CCHF case, supports the absence of CCHFV in Corsica to date. 

In any case, the absence of a documented case of CCHF together with the lack of detection of CCHFV RNA in tick species that are recognized as a competent vector enables us to declare that Corsica is not a hotspot for CCHFV and that the threat to the human population is very limited. However, this discrepant set of data pleads for a One Health approach for dealing with the CCHF question in Corsica and the potential exposure of island population. To do so, the roadmap established by the World Health Organization’s R&D blueprint (https://www.who.int/teams/blueprint/about) should be followed. Because the accuracy of CCHFV serologic assays has been questioned, several tests must be combined as advocated. Then, serologic studies in animals and humans must be synchronized with virus detection in ticks and systematic screening of patients with uncharacterized febrile illness during the tick season. A need exists for a large-scale One Health prospective program for surveillance of ticks, vertebrates, and humans in Corsica.
